# Burnout and Post-traumatic Stress Disorder Symptoms Among Emergency Medicine Resident Physicians During the COVID-19 Pandemic

**DOI:** 10.5811/westjem.2021.11.53186

**Published:** 2022-02-28

**Authors:** Jungsoo Chang, Jessica M. Ray, Daniel Joseph, Leigh V. Evans, Melissa Joseph

**Affiliations:** * Yale School of Medicine, New Haven, Connecticut; † Yale School of Medicine, Department of Emergency Medicine, New Haven, Connecticut

## Abstract

**Introduction:**

Emergency medicine is characterized by high volume decision-making while under multiple stressors. With the arrival of the severe acute respiratory syndrome coronavirus 2 (SARS-CoV-2) virus in early 2020, physicians across the world were met with a surge of critically ill patients. Emergency physicians (EP) are prone to developing burnout and post-traumatic stress disorder (PTSD), due to experiencing emotional trauma as well as the cumulative stress of practice. Thus, calls have been made for attempts to prevent physician PTSD during this current pandemic.

**Methods:**

From July 2019–January 2020, emergency medicine (EM) resident physicians at a large, academic healthcare system were surveyed for symptoms of burnout using the Maslach Burnout Inventory (MBI). In late April and early May 2020, during the outbreak surge of coronavirus disease 2019 (COVID-19) in the Northeast USA, these same residents and the whole EM residency at the institution were again surveyed for symptoms of burnout as well as post-traumatic stress using the PTSD Checklist for Diagnostic and Statistical Manual of Mental Disorders, 5th Edition (PCL-5). A final survey was administered to the EM residents after the COVID-19 surge had largely subsided in June 2020.

**Results:**

Twenty-two residents participated in the pre-pandemic study and completed the MBI. Twelve (55%) completed the two follow-up MBI surveys. In the larger EM residency cohort, 31/60 residents completed the MBI and PCL-5 survey during the pandemic peak and 30/60 (50%) completed the follow-up surveys. There were no significant differences in the three MBI burnout category measures of emotional exhaustion (P = 0.49), depersonalization (P = 0.13), and personal accomplishment (P = 0.70) pre-, during, and post-COVID. Of 31 participants, 11 (35%) scored greater than 31 on the PCL-5. Two residents had scores between 21–30, interpreted as “at risk.” At greater than one month follow-up, 2/30 continued to meet criteria for a preliminary PTSD diagnosis, and five were “at risk.”

**Conclusion:**

A significant proportion of residents (35%) experienced post-traumatic symptoms acutely during the COVID-19 pandemic crisis, potentially indicating a high prevalence of acute stress disorder in this population and increased risk of developing PTSD. However, there was no significant difference in burnout levels in this cohort before, during, or after the initial COVID-19 surge. Early screening for physicians at risk and referral for assessment and treatment may be important to mitigate pandemic-related PTSD.

## INTRODUCTION

Emergency medicine (EM) is characterized by a high volume of decision-making under high stress. Emergency physicians (EP) are particularly prone to developing burnout, with 35–77.8% of physicians reporting significant risks.^1^,^2^ Burnout, characterized by emotional exhaustion, depersonalization, and feelings of decreased personal accomplishment, affects the physicians and their patients.^3^–^6^ Workload, violence, traumatic events, uncontrolled stress, work-family conflict, and poor staffing have been identified as factors in EM that contribute to burnout.^1^,^7^,^8^ Burnout has been associated with lower reported quality of life and of education among residents, as well as increased early retirement and turnover among EPs in practice.^6^,^9^

Emergency physicians are also at increased risk of developing post-traumatic stress disorder (PTSD), both due to experiencing emotional trauma as well as the cumulative stress of practice.^10^,^11^ One study identified 15.8% of EP respondents as meeting preliminary criteria for PTSD, with prior trauma or abuse as the primary predictor.^12^ Post-traumatic stress disorder is characterized by exposure to an extreme stressor or traumatic event followed by at least one month of three distinct types of symptoms: re-experiencing the event; avoidance of reminders of the event; and hyperarousal.^13^ In addition to prior trauma, female gender, genetics, family and personal psychiatric history, impaired executive function, trauma intensity and type, and physiological arousal have been identified as risk factors for development of PTSD after trauma. Social support has been identified as a protective factor.^14^ For EM resident physicians, one institution found that 11.9% met criteria to diagnose PTSD, and that the proportion of residents meeting criteria increased with level of training.^15^ A recent survey of surgical residents found 22% screened positive for PTSD, with an additional 35% considered “at risk.”^16^

With the arrival of the severe acute respiratory syndrome coronavirus 2 (SARS-CoV-2) virus in early 2020, physicians across the world were met with a surge of critically ill patients. From experience in prior pandemics such as SARS in 2003, we know that healthcare workers who treated SARS patients had significantly higher levels of burnout and PTSD, a condition that portends a high risk of suicidal ideations, attempts, and completions.^17^–^20^ Physicians may be particularly at risk, and thus calls have been made for attention to helping to prevent PTSD during this current pandemic.^17^,^21^ Data from China, the first country to experience the pandemic surge, confirms this prediction, with 27% of their medical staff who treated coronavirus disease 2019 (COVID-19) patients reporting post-traumatic stress symptoms.^22^

After the arrival of Sars-CoV-2 to the United States, the Northeast experienced an influx of cases during the months of March, April, and May 2020. (CDC). By May 31, 2020, as the initial surge waned, 42,743 people had been infected with SARS-CoV-2 and 3970 had died. There was an average daily hospital census of 1174 persons statewide.^23^ As the majority of hospital admissions are first managed and stabilized in the emergency department (ED), the volume of COVID-19 patients and its strain on resources was acutely felt by the ED staff. In this study, we examine the association between the peak and wane of COVID-19 cases on physicians at a large, academic healthcare system and symptoms of burnout and post-traumatic stress among our EM resident population. We were able to compare the burnout across a same matched cohort across three time periods: before, during, and after the peak COVID surge in the Northeast US to better elucidate the effectiveness of current methods to reduce physician burnout.

**Population Health Research Capsule**

What do we already know about this issue?

*Emergency physicians are prone to burnout and post-traumatic stress disorder (PTSD), due to emotional trauma and the cumulative stress of practice.*

What was the research question?

*With coronavirus disease 2019 (COVID-19), was there a change in burnout and PTSD symptoms among the emergency medicine resident physicians?*

What was the major finding of the study?

*Our study population had no change in burnout during or after the COVID-19 surge in the Northeast US but had increased symptoms of PTSD.*

How does this improve population health?

*Most residents’ PTSD symptoms had improved at follow-up. Early screening for physicians at risk and referral for treatment may help mitigate pandemic-related PTSD.*

## METHODS

### Study Design

This was a prospective, cohort longitudinal study of EM resident physicians before and during the COVID-19 pandemic. This study was determined to be exempt by the institutional review board.

### Study Setting and Population

From July 2019–January 2020, EM resident physicians (postgraduate years [PGY] 1–4) at a large, academic healthcare system were surveyed for symptoms of burnout during their elective rotation. We had pre-COVID Maslach Burnout Inventory Human Services Survey for Medical Personnel (MBI-HSS (MP) data from EM resident cohorts that had rotated through the simulation rotation up to the point of the pandemic outbreak. In late April and early May 2020, during the outbreak surge of COVID-19 in the Northeast, a sample of residents from this same resident cohort were again surveyed for symptoms of burnout and post-traumatic stress as they worked in clinical areas. A final survey was administered to the entire EM residency cohort after the COVID-19 surge had largely subsided in June 2020. The EM residents in this institution worked solely in the ED, without off-service rotations, from April 7–July 1, 2020.

During the initial survey period, there were no reported cases of COVID-19 in the US. On data collection days during the COVID-19 surge from late April to mid-June 2020, there were 50 average daily admissions, an average daily hospital census of 634 patients, and 153 patients in the intensive care unit with COVID-19 throughout this academic healthcare system. During the final survey in late June 2020, there was an average of six daily COVID-19 admissions, and a daily census of 52 admitted patients with COVID-19 across the healthcare system, with 26 at the primary teaching site.

### Study Protocol

Resident physicians completed a MBI-HSS (MP)) as part of a separate study and results were de-identified. During the COVID-19 pandemic, a follow-up MBI-HSS (MP), as well as the PTSD Checklist (PCL-5) for the *Diagnostic and Statistical Manual of Mental Disorders, 5^th^ Edition* (*DSM-5*) were distributed to this cohort as well as the larger residency cohort via anonymous survey using Qualtrics software (Qualtrics, Provo, UT). An identical survey was again distributed after the initial COVID-19 surge subsided in the Northeast. Responders were matched between the second and third surveys via a unique self-identifying code. Responders to the second and third surveys were also asked to indicate whether they had participated in the initial, pre-COVID-19 MBI-HSS (MP) survey so that results could be compared longitudinally within this smaller cohort. For the initial survey, written consent was obtained in person. For the follow-up surveys, written consent was obtained electronically. With the second and third surveys, participants were provided with a list of resources for support during the pandemic, both departmentally and institutionally.

### Outcome Measures

The primary outcome measures of this study were the scored differences between sequential MBI-HSS (MP) and PCL-5 surveys. The MBI-HSS (MP) is a version of the Maslach Burnout Inventory that was developed for healthcare professionals who have direct contact with patients. The MBI has been validated in many different populations, including healthcare professionals and is the most widely used survey to measure burnout. It is scored in three categories: emotional exhaustion (EE); depersonalization (DP); and personal accomplishment (PA), with high levels of EE and DP and low levels of PA characteristic of burnout.^24^–^26^ While there is no cutoff score that represents a diagnosis of burnout, each category score is interpreted by its frequency of symptoms, which ranges from *Never* (0) to *Every Day* (6).^26^

The PCL-5 is one of the most widely used self-report measures of PTSD, and respondents indicate how much they have been bothered by each PTSD symptom over the prior month using a five-point Likert scale ranging from 0 to 4 with 0 (*not at all*) to 4 (*extremely*).^27^ The item scores are summed to yield a continuous measurement indicating PTSD symptom severity (range 0–80). The PCL-5 is a revised version of the PCL, reflecting the initial *DSM-5* criteria changes for PTSD.^28^ While the PCL-5 score has not yet had extensive cut-off score evaluation for PTSD symptomatology, a PCL-5 score of 31–33 or greater predicts PCL scores of > 40s, a previously established cutoff score that suggests the participant may benefit from PTSD treatment if they meet other diagnostic criteria such as time since stressor. Any item rated 2 (*moderately*) or higher can be considered as a PTSD symptom endorsed based on *DSM-*5.^27^–^29^

We used the reported hospital systemwide COVID inpatient volume as a proxy for the volume and burden of SARS-CoV-2 spread within the state and relative cases seen in the ED. In-patient COVID-19 positive numbers were reported beginning mid-March and have been continuously updated within the system.

### Data Analysis

The responses from the initial survey period (July 2019–January 2020), COVID-19 peak period (April–May 2020), and post-surge period (July 2020) were collected. We calculated a one-way analysis of variance (ANOVA) with R 3.6.2 (R Foundation for Statistical Computing, Vienna, Austria) to determine whether there was a statistically significant difference in burnout symptom categories (EE, DP, and PA) between the three time periods surrounding the COVID pandemic. Both a paired (N = 15 for individuals who had filled both the second and third surveys) and unpaired (N = 28 and N = 30 for second and third surveys, respectively) two-sided t-test were performed on the results from the latter two MBI surveys to determine statistically significant differences in measured burnout categories during peak COVID-19 volume at our site and again in late June. Similarly, paired and unpaired two-sided t-tests were performed on the PCL-5 results using R.

Using the academic healthcare system’s COVID-19 day-to-day inpatient monitor, we used R to determine whether a correlation between in-patient COVID-19 positive rates and MBI or PCL-5 scores existed.

## RESULTS

**Figure 1:**
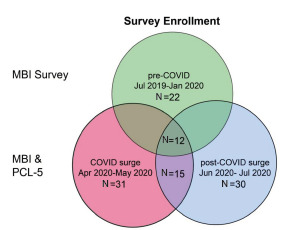
Venn diagram illustrating the survey enrollment population and the surveys taken by each cohort. *MBI*, Maslach Burnout Inventory; *PCL*, post-traumatic stress disorder checklist; *COVID*, coronavirus disease 2019.

Peak COVID-19 inpatient volume occurred in mid-April, with 787 COVID positive patients across our academic healthcare system. After this peak, there was a gradual downward trend in both COVID-19 positive inpatients and new admissions. Twenty-two residents who were in an elective rotation participated in the pre-pandemic study and completed the MBI at that time. Twelve of these residents (55%) completed the sequent two follow-up MBI surveys. In the larger cohort, 31/60 residents completed the MBI and PCL-5 survey during the pandemic peak, and 30/60 completed the follow-up survey, indicating a follow-up response rate of approximately 50% (Figure 1). Fifteen of these residents completed both the intra- and post-pandemic surveys (Table 1).

### Maslach Burnout Inventory-HSS

**Figure 2:**
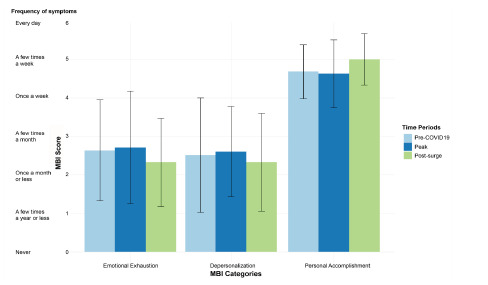
Paired outcomes based on Maslach Burnout Inventory categories (emotional exhaustion, depersonalization, and personal accomplishment) across three different time periods surrounding the first COVID-19 outbreak. The corresponding symptom description is on the y axis corresponding to the Maslach Burnout Inventory (MBI) score.

There was no significant difference in burnout category measures of EE (unbalanced one-way ANOVA *P* = 0.49), DP (*P* = 0.13), and PA (*P* = 0.70) pre-, during, and post-COVID (Figure 2).

MBI-HSS scores from the smaller cohort of individuals who completed the surveys during the peak and post-surge (n=15, paired two sided t-test) were consistent with that of the larger cohort for the latter two time points where there was no significant difference in the burnout category measures for EE (p=0.05516), D (p=0.825), and PA (p=0.7474). There was also no statistically significant difference in burnout categories for all the participants of the survey during the peak and post-surge (EE p=0.2667; D p=0.3859; PA p=0.07574). Additionally, there was no correlation found between MBI-HSS scores and inpatient COVID-19 volume.

### Post-traumatic Stress Disorder Checklist-5

**Figure 3:**
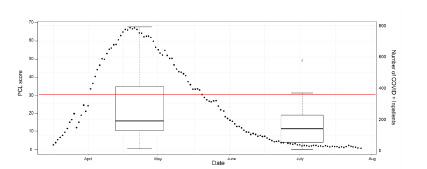
Scatterplot of inpatient COVID-19 patients based on the corresponding dates (right side y axis) with box plot of PCL-5 score during the two survey periods, with box plot width corresponding to the survey administration period. A threshold at 31 is depicted for the PCL-5 score to demonstrate positive screening for PTSD symptoms

Of the surveyed 31 participants, 11 EM residents (35%) scored greater than 31 on the PCL-5, which has a maximum possible score of 80. An additional two residents had scores between 21–30, which was interpreted as “at risk.” At >1 month follow-up, 2/30 respondents continued to meet criteria for a preliminary PTSD diagnosis, and five were classified as “at risk.” Figure 3 shows a boxplot of PCL-5 scores of EM residents throughout the study period with a scatterplot of the number of COVID-19 positive patients, with the width of boxplot corresponding to the dates the surveys were collected (Figure 3).

**Figure 4:**
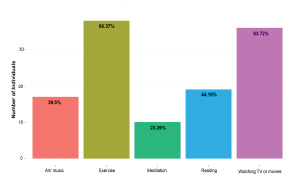
Bar graph depicting how residents reported managing stress across the study period. Number of individuals is on the y axis, and percentage of total residents participating in the activity is written in each bar. *EM*, emergency medicine.

A threshold line of 31 on the box plot represents the cutoff score of the PCL-5. There was a statistically significant difference in PCL-5 values during these two study periods. Peak COVID surge PCL-5 mean was 22 while post-surge was 13 (N = 28 for peak surge and N = 30 for post-surge, unpaired two-sample t-test *P* = 0.03). When comparing the smaller group of paired individuals (N = 15), there was no statistically significant difference in PCL-5 values (paired t-test *P*-value = 0.14), with a mean of 22 during and 14 post COVID-19 surge volumes. A majority of residents reported using exercise and watching TV or movies as tools to help manage stress (88.4%, 83.7% respectively). A minority of residents reported engaging in art, music, meditation, and/or reading (Figure 4).

## DISCUSSION

Attention to physician mental health is perhaps now more than ever imperative. The stressor of the ongoing pandemic, laid on top of a limitation in resources, public skepticism of the disease, and a field that is mentally taxing at baseline puts our healthcare clinicians at increased risk for crisis. This is highlighted by the death of Dr. Lorna M. Breen, who died by suicide while in the midst of treating COVID-19 victims as the medical director of an ED in Manhattan, New York City, NY.^30^

Our work did not show a significant change in burnout symptom frequency pre-, intra-, and post-COVID-19 surge among the EM residents, which was unexpected. Possible reasons for a lack of change include work hours limitations, higher baseline burnout among resident physicians, or duration of the pandemic peak, and perceived increase of public opinion of healthcare workers and emergency physicians in particular, among others, although our small sample size may also be masking significant findings. Additionally, all residents practice under a supervising physician, and deliberate changes within the department limited exposure of junior residents to very ill COVID-19 patients. For example, only PGY-3 and PGY-4 residents were involved in intubations or resuscitation of unstable, suspected COVID-19 persons under investigation at the primary teaching site. Further studies within the institution and across the country examining burnout among healthcare personnel at large are forthcoming and should help to elucidate some of these factors.

Our cohort of residents did experience a significant number of PTSD symptoms related to COVID-19 during the peak volume, with two residents having persistent symptoms sufficient to make a preliminary diagnosis of PTSD at and a further five just under the defined threshold. While physicians have a higher baseline level of PTSD, 35% of our participants screened positive based on the PCL-5, which is far higher than the prevalence found in prior studies. The significant decrease in scores on the follow-up survey is consistent with existing knowledge that many persons who experience initial acute stress disorder symptoms do not progress to PTSD, although they are at higher risk.^13^ Interestingly, in our smaller cohort, PTSD symptoms did not decrease significantly, which suggest more persistent symptoms in this subgroup. With the number of rising cases across the country again, it is crucial to identify the physicians at risk for development of PTSD and work toward prevention and treatment of those who are affected.

Post-traumatic stress disorder has an identifiable onset and symptom profile, making it accessible for early diagnosis and intervention. Cognitive behavioral therapy (CBT) is the current mainstay of treatment. The therapy can involve different approaches and aims, such as exposure-based treatment, aimed at controlling reactions and decreasing avoidance, and cognitive-based CBT, which is aimed at challenging beliefs surrounding meaning and implications of the trauma using available evidence and guidance.^31^,^32^ For physicians with PTSD symptoms it is thus imperative that early recognition and referral are priorities of programs and care aimed at helping this population.

## LIMITATIONS

Our study is limited foremost by sample size. We felt that it was important that we had pre-COVID MBI-HSS data, but this limited our participants to the resident cohort that had rotated through their simulation rotation up to the point of the pandemic outbreak. We found that our MBI results were similar to a larger, multicenter study of 261 EM residents, suggesting our smaller cohort is representative of a typical EM program.^33^ Our overall follow-up response rate was approximately 50%. Informally, we received feedback that the residents had survey fatigue during this time, with several ongoing study initiatives during the pandemic in addition to clinical duties amounting to a palpably increased overall workload.

We found that 35% of our participants screened positive for PTSD symptoms related to the COVID-19 pandemic using the PCL-5. This instrument cannot, however, be used in isolation as a diagnostic tool. A formal diagnosis of acute stress disorder and/or PTSD would rely on a structured diagnostic interview, which was not done in this study. Additionally, further work would be needed to compare underlying risk factors of our cohort and COVID-19 volume, institutional support, case severity, and other factors with EM residents at large to inform generalizability of our findings across programs. Our paired cohort had a decrease in PCL-5 scores that did not reach significance, which we suspect may be secondary to a lower N but could also be due to more persistent symptoms in this subgroup.

## CONCLUSION

Our resident physician study population did not experience a significant change in burnout symptoms from pre-COVID-19 baseline during or following the case surge in Northeast US. A substantial proportion of residents (35%) experienced post-traumatic stress symptoms acutely during the crisis, potentially indicating a high prevalence of acute stress disorder in this population and increased risk of developing PTSD. The majority of residents had improvement of these symptoms at a greater than one month follow-up. Future work in delineating factors in burnout and PTSD in healthcare personnel may be interesting given these findings. Early screening for physicians at risk and referral for assessment and treatment will be crucial to help mitigate pandemic-related PTSD. Strategies for early detection and primary prevention of PTSD among physicians is ripe for further study, with the most important factors being early identification and referral.

## Figures and Tables

**Table 1:** Maslach Burnout Inventory for medical personnel category outcomes based on pre-, intra-, and post COVID-19 time periods.

	Emotional Exhaustion	Depersonalization	Personal Accomplishment
Pre- COVID 19 (N = 22)			
Mean	2.64	2.51	4.68
SD	1.31	1.49	0.7
Intra- COVID 19 (N = 28)			
Mean	2.74	2.57	4.61
SD	1.47	1.15	0.84
Post- COVID 19 (N = 30)			
Mean	2.33	2.33	5.00
SD	1.14	1.27	0.67

*COVID-19*, coronavirus disease; *SD*, standard deviation.
